# Expression of Recombinant Hirudin in Bacteria and Yeast: A Comparative Approach

**DOI:** 10.3390/mps8040089

**Published:** 2025-08-03

**Authors:** Zhongjie Wang, Dominique Böttcher, Uwe T. Bornscheuer, Christian Müller

**Affiliations:** 1 Zoological Institute and Museum, Department of Animal Physiology, University of Greifswald, 17489 Greifswald, Germany; zhongjiewang528@gmail.com; 2 Institute of Biochemistry, Department of Biotechnology and Enzyme Catalysis, University of Greifswald, 17489 Greifswald, Germany; dominique.boettcher@uni-greifswald.de (D.B.); uwe.bornscheuer@uni-greifswald.de (U.T.B.)

**Keywords:** hirudin, hirudin-like factor, *Escherichia coli*, *Komagataella phaffii*, recombinant protein expression

## Abstract

The expression of recombinant proteins in heterologous hosts is a common strategy to obtain larger quantities of the “protein of interest” (POI) for scientific, therapeutic or commercial purposes. However, the experimental success of such an approach critically depends on the choice of an appropriate host system to obtain biologically active forms of the POI. The correct folding of the molecule, mediated by disulfide bond formation, is one of the most critical steps in that process. Here we describe the recombinant expression of hirudin, a leech-derived anticoagulant and thrombin inhibitor, in the yeast *Komagataella phaffii* (formerly known and mentioned throughout this publication as *Pichia pastoris*) and in two different strains of *Escherichia coli*, one of them being especially designed for improved disulfide bond formation through expression of a protein disulfide isomerase. Cultivation of the heterologous hosts and expression of hirudin were performed at different temperatures, ranging from 22 to 42 °C for the bacterial strains and from 20 to 30 °C for the yeast strain, respectively. The thrombin-inhibitory potencies of all hirudin preparations were determined using the thrombin time coagulation assay. To our surprise, the hirudin preparations of *P. pastoris* were considerably less potent as thrombin inhibitors than the respective preparations of both *E. coli* strains, indicating that a eukaryotic background is not per se a better choice for the expression of a biologically active eukaryotic protein. The hirudin preparations of both *E. coli* strains exhibited comparable high thrombin-inhibitory potencies when the strains were cultivated at their respective optimal temperatures, whereas lower or higher cultivation temperatures reduced the inhibitory potencies.

## 1. Introduction

Hirudin of the medicinal leech, *Hirudo medicinalis* Linne (1758), is a small molecule of about 7 kDa in size [1]. It is a very strong natural inhibitor of thrombin and hence a very effective anticoagulant [2]. In fact, it is so far the one and only leech-derived bioactive factor that found its way from “bench to bedside” [3,4,5]. The structure of hirudin is characterized by the presence of six conserved cysteine residues that form three intra-molecular disulfide bonds and thereby stabilize the so-called central globular domain [6,7]. The thrombin-inhibitory effect of hirudin depends on both the short N-terminus and the elongated C-terminal tail of the molecule. The N-terminal five amino acid residues of hirudin penetrate the active-site cleft and block the catalytic center of thrombin, whereas the C-terminal tail binds to exosite 1, the fibrinogen-binding site of thrombin [8]. Hirudin is hence a bivalent thrombin inhibitor [9]. However, the thrombin-inhibitory effect of hirudin is also strongly influenced by the central globular domain, very likely through the correct spacing, positioning and orientation of both termini and by stabilizing the binding to thrombin [10]. Several variants of hirudin were described in *H. medicinalis*, including variant HV1 (VV-hirudin), variant HV2 (IT-hirudin) and two forms of variant HV3 (PA-hirudin) [1,11,12]. All variants exhibit approximately the same thrombin-inhibitory potencies [13].

Hirudin-like factors (HLF) represent an additional group of leech-derived bioactive factors. They exhibit certain shared features with hirudins including six cysteine residues within the central globular domain and a conserved gene structure that comprises four exons and three introns [13]. However, besides common features, hirudins and HLFs significantly deviate in other aspects, such as in their molecular masses and isoelectric points (pI values) [14]. While certain HLFs (including HLF1V) display thrombin-inhibitory potencies equivalent to those of hirudins, others demonstrate either minimal or no thrombin inhibition [13,15,16].

Living medicinal leeches of several genera are used for bloodletting and the treatment of circulatory disorders since ancient times [17,18]. In addition, dried leeches (Shui Zhi) are an ingredient of Traditional Chinese Medicine (TCM) and are routinely orally administered to cure blood stasis and to stimulate menstruation discharge [19,20]. In modern Western medicine, hirudin is clinically used as an alternative to heparin in patients that suffer from heparin-induced thrombocytopenia (HIT), a potentially lethal complication of long-term heparin administration [21,22]. Both yeast-based and bacterial expression systems are commonly used for the production of recombinant hirudin [11,23,24,25], and both alternatives lead to hirudin preparations that are almost as equally effective as natural hirudin [26]. However, only recently, the cell-free synthesis of hirudin HV1 was shown to be a convincing alternative to the recombinant expression, yielding a form of hirudin HV1 that exhibited a thrombin-inhibitory potency of up to 100 times higher than bacterially expressed hirudin HV1 [27]. This observation highlights the necessity to further evaluate and improve the “classical” expression strategies for hirudin. Here we present an investigation that compares a yeast-based eukaryotic expression system and two bacterial expression systems in terms of the thrombin-inhibitory potencies of the respective recombinant hirudin preparations.

## 2. Materials and Methods

### 2.1. Expression of Recombinant Hirudin HV1 and HLF1V in E. coli

For expression, pQE30Xa clones (Qiagen, Hilden, Germany) containing cDNAs encoding hirudin HV1 (GenBank accession number KR066903.1; [13]) or HLF1V (GenBank accession number KR066909.1; [10,13]) of *H. medicinalis* were transformed into either *E. coli* DH5α cells (Thermo Scientific, Darmstadt, Germany; [28]) or SHuffle^®^ T7 cells (New England Biolabs, Frankfurt a. M., Germany; [29]) using chemically competent cells and the standard procedure described by Green and Sambrook [30]. Cells were plated out on LB (lysogeny broth; 5 g of NaCl, 10 g of tryptic peptone ex casein and 5 g of yeast extract per liter of Aqua dest) agar plates containing 100 μg/mL of ampicillin for selection and incubated overnight for 16–20 h at 37 °C. A few colonies were used to inoculate an overnight culture of a final volume of 11 mL of LB medium containing 100 μg/mL of ampicillin and shaken at either 37 °C (strain DH5α) or 30 °C (strain SHuffle^®^ T7). The next morning, two flasks, each containing 500 mL of LB medium with ampicillin, were inoculated with 5 mL of the overnight culture and incubated under constant shaking at 30 °C (experiment 1) or at 22, 30, 37 and 42 °C, respectively (experiment 2). Optical densities were determined in a regular manner using a DU530 spectrophotometer (Beckman Coulter, Krefeld, Germany). At OD_600_ = 0.5, the expression of recombinant hirudins was induced by adding isopropyl-β-D-thiogalactopyranoside (IPTG) to a final concentration of 1 mmol/L.

After 3 h of incubation, the cells were harvested (centrifugation for 20 min at 4 °C and 3900× *g*; Labofuge 400R, Thermo Scientific, Schwerte, Germany), the pellets were carefully resuspended in 25 mL of binding buffer (20 mmol/L of Tris/HCl, 500 mmol/L of NaCl and 5 mmol/L of imidazole; pH 7.9) and the cells were sonicated using a Sonoplus homogenizer (Bandelin, Berlin, Germany) by applying a total of 10 sonication cycles with the following parameters: 75 impulses with a duration of 0.8 s and a power of 65–68% each. To avoid heating, the samples were placed on ice during sonication and cooled on ice for 2 min before the start of the next cycle. After sonication, the samples were centrifuged for 45 min at 4 °C and 3900× *g* (Labofuge 400R, Thermo Scientific, Schwerte, Germany). The supernatant was carefully removed and centrifuged for an additional 10 min under the same conditions.

### 2.2. Expression of Recombinant Hirudin HV1 in P. pastoris

For the expression of recombinant hirudin HV1 in *P. pastoris*, a respective vector based on pPIC9K (Invitrogen^TM^, Thermo Scientific, Schwerte, Germany) was constructed using the gene synthesis service of Biocat (Biocat GmbH, Heidelberg, Germany). The vector confers resistance to kanamycin in bacteria and G-418 (geneticin^TM^) in eukaryotic cells, secures a stable integration into the genome of *P. pastoris* and allows for the expression of recombinant proteins under the control of the methanol-inducible alcohol oxidase 1 (AOX1) promoter. In addition, it contains an alpha factor secretion signal to guide the expressed recombinant protein to the growth medium. The cDNA sequence of hirudin HV1 that was used to construct the yeast expression vector was the same as was used for the expression in bacterial cells (see above) and hence, contained the coding regions for both a His-tag and a factor Xa protease cleavage site, located 5′ to the coding region of hirudin HV1.

*Sac*I-linearized plasmids were introduced into competent cells of *P. pastoris* strain GS115 (Invitrogen^TM^, Thermo Scientific, Schwerte, Germany) by electroporation using the following settings: 400 V/cm, 10 μF, low resistance. Transformed cells were selected on minimal dextrose medium (MD) plates. Final clones were selected by a two-step-process: (1) transferring single colonies from MD to YPD plates with increasing concentrations of geneticin (1 mg/mL, 1.5 mg/mL and 1.75 mg/mL, respectively) and (2) transferring single colonies from YPD plates to minimal methanol (MM) plates. Two clones (2 and 29) were selected for the following experiments.

Both clones were first inoculated in 5 mL of buffered glycerol complex (BMGY) medium and incubated overnight at 30 °C and under constant shaking. Then, 1 mL of the overnight cultures was used to inoculate 50 mL of fresh BMGY medium. The cultures were incubated at 30 °C under constant shaking and the optical densities were measured in a regular manner. At OD_600_ = 6, the cells were harvested, resuspended in 10 mL of buffered methanol complex (BMMY) medium, and 1 mL of the resuspended cell solution was used to inoculate 500 mL of fresh BMMY medium. The cultures were incubated for an additional four days at 30 °C (experiment 1) or 20 °C (experiment 2) under constant shaking, and each day 2.5 mL of methanol was added.

At the end of cultivation, the cells were harvested by centrifugation (Labofuge 400R, 45 min at 4 °C and 3900× *g*) and the supernatants containing the secreted His-tagged hirudin HV1 were transferred to 50 mL tubes (Sarstedt, Nümbrecht, Germany). To each tube containing 45 mL of supernatant the following components were added: 5 mL of 200 mmol/L Tris/HCI, pH 7.9 (final concentration 20 mmol/L); 1.461 g of NaCl (final concentration 500 mmol/L) and 0.5 mL of a 500 mmol/L imidazole solution (final concentration 5 mmol/L). In addition, the cell pellets were resuspended in binding buffer and sonicated as described above.

### 2.3. Purification of Recombinant Hirudins HV1 and HLF1V

The supernatant after sonication of the bacterial cells or the supernatant after the cultivation of *P. pastoris* was loaded onto a self-packed column containing 2 mL of Ni-NTA His-Bind^®^ resin (Merck, Darmstadt, Germany). Washing and elution steps were performed as recommended by the manufacturer of the resin. In detail, a stepwise washing and elution protocol was used with buffers (20 mmol/L Tris/HCl and 500 mmol/L NaCl; pH 7.9) containing increasing concentrations of imidazole (20 mmol/L, 50 mmol/L, 100 mmol/L, 200 mmol/L and 500 mmol/L, respectively). A final strip buffer (100 mmol/L EDTA) was applied to completely remove the Ni ions from the column. All elution fractions were collected, and equal volumes of every fraction were analyzed by SDS-PAGE on 20% acrylamide gels. Gel images were generated using a Gel Doc EZ imager (BioRad, Feldkirchen, Germany) with an automatic internal algorithm to adjust the exposure time of the images. Prior to the factor Xa protease treatment, fractions of interest were dialyzed (regenerated cellulose dialysis membrane Spectra/Por^®^ 7, MWCO 3.5 kDa; Carl Roth GmbH, Karlsruhe, Germany) two times for 24 h at 4 °C against a 100-fold excess of reaction buffer (20 mmol/L Tris/HCl, 50 mmol/L NaCl and 1 mmol/L CaCl_2_; pH 8.0).

Treatment of fusion proteins containing the factor Xa protease recognition sequence consisted of two steps: (1) factor Xa protease cleavage and (2) removal of factor Xa protease. Both steps were performed as recommended by the manufacturer (New England Biolabs, Frankfurt a. M., Germany). In total, 1.7 mL of each hirudin HV1 or HLF1V preparation was incubated with 3 µg of factor Xa (3 μL of a 1 mg/mL stock solution) for 72 h at 22 °C in reaction buffer. The success of the factor Xa treatment was controlled by SDS-PAGE analysis. Factor Xa protease was removed by adding an appropriate volume of equilibrated Xarrest^TM^ Xa protease removal resin (Merck, Darmstadt, Germany) to the reaction mixtures, incubating for 10 min at room temperature under constant shaking and collecting the supernatant following a centrifugation step for 5 min at 1000× *g*.

Molar concentrations of the recombinant hirudin HV1 and HLF1V solutions were calculated by measuring the absorbance at λ = 280 nm and dividing the obtained values by the molar absorption coefficient according to the equation ε = (nW × 5500) + (nY × 1490) + (nC × 125) [31,32].

### 2.4. Blood Coagulation Assays

To verify the thrombin-inhibitory potencies of all recombinant hirudin HV1 and HLF1V preparations, we applied the thrombin time test (TT; reference range 16.8–21.4 s) using a BFT II analyzer (Siemens Healthcare, Erlangen, Germany). The TT test specifically addresses the final step of the coagulation cascade—the thrombin-mediated conversion of fibrinogen to fibrin—and is hence, very sensitive to direct thrombin inhibitors like hirudin. The test optically and mechanically determines the formation of a fibrin clot and measures the time upon clot formation. All steps were carried out according to the instructions of the manufacturer. Protein samples were diluted with reaction buffer to reach the final concentrations of 3.2 µmol/L, 0.32 µmol/L or 0.032 µmol/L in the reaction assays. The desired amounts of thrombin reagent (50 µL of bovine thrombin, Siemens Healthcare, Erlangen, Germany) and test substrate (10 µL of buffer, hirudin HV1 or HLF1 preparations, respectively) were directly transferred into the test cuvette immediately before 100 µL of standardized human plasma (Dade^®^ Ci-Trol^®^ 1, Siemens Healthcare, Erlangen, Germany) was added. The incubation of the reaction mixtures was carried out at 37.4 °C and the time until the formation of a fibrin clot was measured. Measurements that exceeded 300 s were stopped and considered as the complete inhibition of clot formation.

## 3. Results

### 3.1. Expression of Recombinant Hirudin HV1 and HLF1V in Escherichia coli Strains

**Figure 1:**
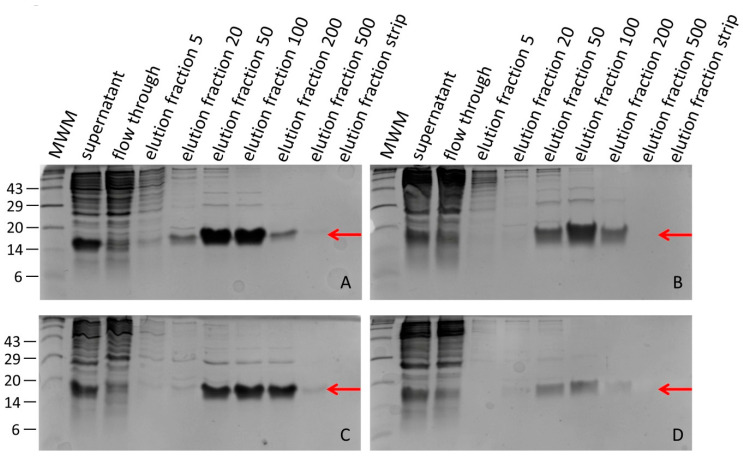
SDS-PAGE analysis of elution fractions of hirudin HV1 (**A**,**C**) and HLF1V (**B**,**D**) after expression in *E. coli* strains DH5α (**A**,**B**) and SHuffle^®^ T7 (**C**,**D**). The red arrows indicate the respective factors. The numbers on the left indicate the molecular mass, in kDa, of the respective molecular weight marker (MWM) bands. The numbers 5–500 indicate the imidazole concentrations in mmol/L in the respective elution fractions.

Hirudin HV1 and HLF1V were expressed in the *E. coli* strains DH5α and SHuffle^®^ T7 at 30 °C and were present in the soluble cytosolic fraction. Both factors could be successfully purified by His-tag affinity chromatography. SDS-PAGE analysis revealed that hirudin HV1 was eluted over a broad range of imidazole concentrations, between 20 mmol/L and 500 mmol/L, with almost equal amounts of recombinant protein present in the 50 mmol/L and 100 mmol/L imidazole elution fractions, whereas HLF1V was eluted over a more narrow range of imidazole concentration, between 50 mmol/L and 200 mmol/L, with a sharp peak in the 100 mmol/L imidazole fraction (Figure 1).

The molar concentrations of hirudin HV1 or HLF1V in the respective elution fractions were determined as described in Section 2 and used to calculate the total yields of recombinant proteins; the results are summarized in Table 1.

### 3.2. Processing Recombinant Hirudin HV1 and HLF1V

**Figure 2:**
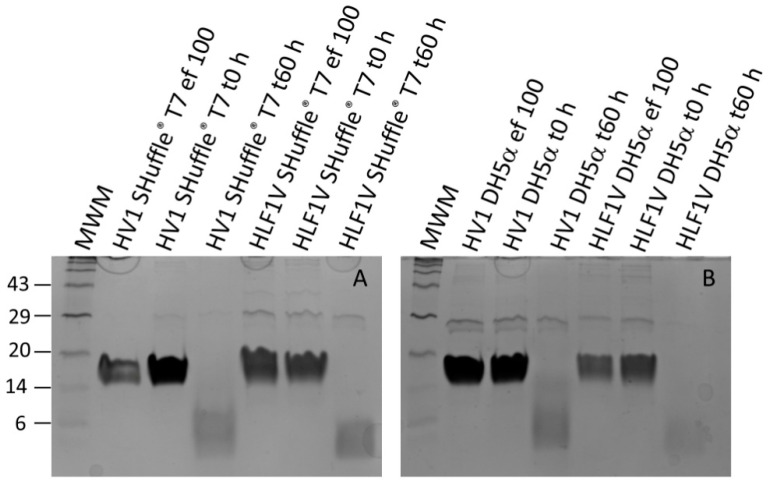
Factor Xa protease treatment of selected elution fractions (ef) of hirudin HV1 and HLF1V expressed in *E. coli* strains SHuffle^®^ T7 (**A**) or DH5α (**B**). The numbers on the left indicate the molecular mass, in kDa, of the respective molecular weight marker (MWM) bands.

In all cases the 100 mmol/L imidazole elution fractions contained only little amounts of unspecific contamination (see Figure 1), and these were selected for further processing. After dialysis, the selected hirudin HV1 and HLF1V fractions were subject to a factor Xa treatment for 60 h to remove the N-terminal His-tag. The complete removal of the His-tag was confirmed by subsequent SDS-PAGE analyses (Figure 2).

Please note that untagged hirudin HV1 and HLF1V are very poorly stained by Coomassie brilliant blue stain due to the low number (hirudin HV1) or even absence (HLF1V) of basic amino acid residues in the molecules, whereas the His-tagged proteins are properly stained due to the His-tag that not only has six histidine residues but also two additional basic amino acid residues.

After the removal of factor Xa protease (see Section 2), the protein concentrations in all treated samples were determined. The values differed only slightly and were in a range of about 130–190 µmol/L or 840–1340 µg/mL (see Table 2 for details).

### 3.3. Functional Characterization of Hirudin HV1 and HLF1V Expressed in E. coli Strains

**Figure 3:**
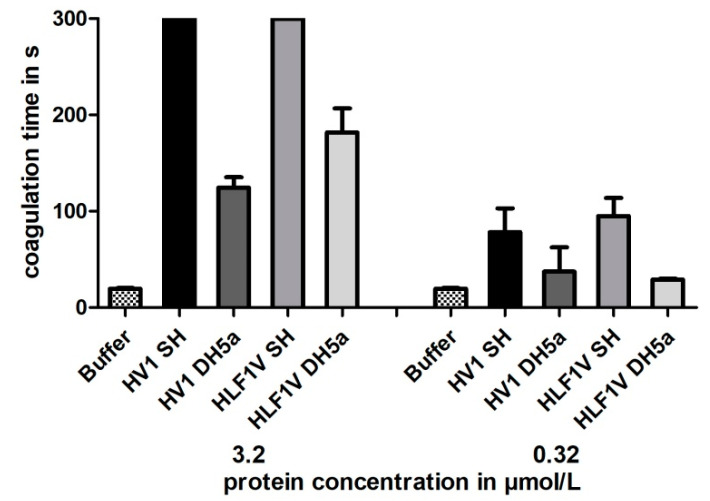
Thrombin time coagulation assays of recombinant hirudin HV1 and HLF1V preparations after expression in *E. coli* strains SHuffle^®^ T7 (SH) and DH5α. Results are means of three independent measurements.

All samples were subject to a functional characterization by the Thrombin Time Test (TT-Test), a coagulation assay that specifically addresses the activity of thrombin. The final concentrations of hirudin HV1 and HLF1V were adjusted to 3.2 µmol/L and 0.32 µmol/L, respectively; the values thereby followed our previous investigations. All samples displayed a strong thrombin-inhibitory potency, but only the preparations of hirudin HV1 and HLF1V out of *E. coli* strain SHuffle^®^ T7 reached the cut-off value of 300 s in the higher final concentration of 3.2 µmol/L. The preparations of both factors out of *E. coli* strain DH5α were noticeably less potent as thrombin inhibitors (see Figure 3).

**Figure 4:**
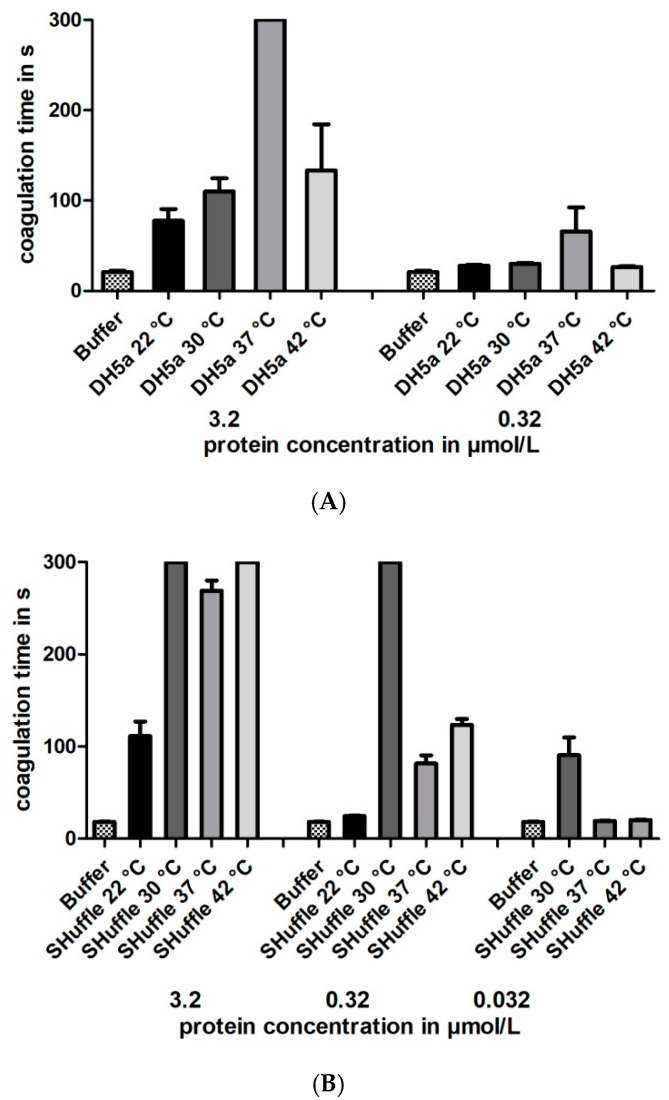
Thrombin time coagulation assays of recombinant hirudin HV1 preparations after cultivation and expression in *E. coli* strains DH5α (**A**) or SHuffle^®^ T7 (**B**) at 22, 30, 37 or 42 °C, respectively. Results are means of three to six independent measurements.

The optimal cultivation temperature of *E. coli* strain DH5α is 37 °C, whereas the optimal cultivation temperature of *E. coli* strain SHuffle^®^ T7 is 30 °C. To evaluate whether the cultivation temperature might influence the thrombin-inhibitory potencies of hirudin HV1 preparations, the bacteria of both strains were cultivated and the proteins expressed at the following different temperatures: 22, 30, 37 and 42 °C, respectively. The experimental routines were the same as described above. The cultivation parameters including the optical densities at start of expression and harvesting are provided in Table S1. All factors were purified, and the total yields (see Table S2) and the final concentrations of the processed recombinant proteins (see Table S3) were in approximately the same range as for the preparations of the first expression experiment. All factors were functionally characterized, and the results clearly showed that the maximal inhibitory potency of hirudin HV1 was only achieved when the respective strains were cultivated at their optimal temperatures (37 °C for DH5α and 30 °C for SHuffle^®^ T7), whereas lower or higher cultivation temperatures led to hirudin HV1 preparations of reduced inhibitory potencies (see Figure 4 for details).

### 3.4. Expression of Recombinant Hirudin HV1 in P. pastoris GS115

As described in Section 2, the recombinant hirudin HV1 that was expressed in *P. pastoris* GS115 contained a factor alpha secretion signal followed by the His-tag and the mature hirudin protein. We applied this strategy to achieve comparability with the His-tag purification process after bacterial protein expression (see above). Two clones, namely clone 2 and clone 29, were selected and cultivated as already described. Protein expression experiments in *P. pastoris* are routinely performed at temperatures up to 30 °C, but lower temperatures may reduce cell stress and enhance productivity [33]. We decided to cultivate both clones at 20 and 30 °C.

**Figure 5:**
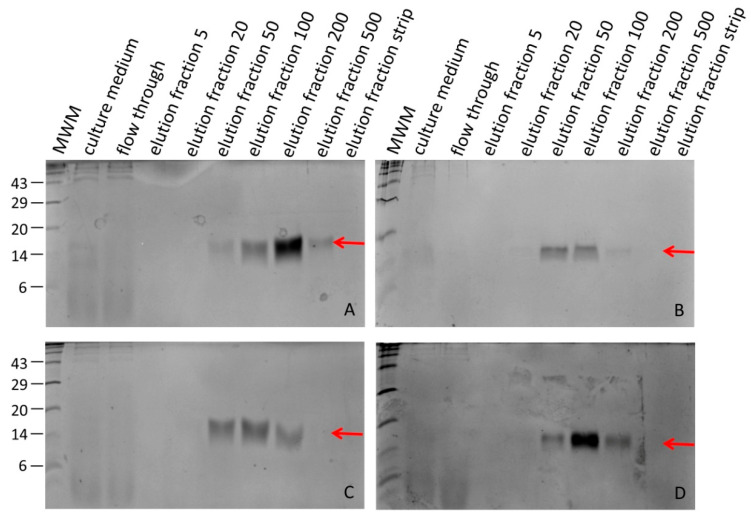
SDS-PAGE analyses of elution fractions of hirudin HV1 after expression in *P. pastoris* GS115 clone 2 (**A,B**) and clone 29 (**C,D**) cultivated at either 20 (**A,C**) or 30 °C (**B,D**). HLF1V (**B,D**). The red arrow marks the HV1 protein. The numbers on the left indicate the molecular mass, in kDa, of the respective molecular weight marker (MWM) bands. The numbers 5–500 indicate the imidazole concentrations in mmol/L in the respective elution fractions.

The culture medium (that should contain the secreted His-tagged hirudin HV1 protein), the cell lysates after sonication and the separated supernatants as well as the resuspended pellets were analyzed by SDS-PAGE to verify the success of protein expression and to identify the fractions that contained the expressed hirudin HV1. However, in none of the fractions could a protein of the expected size be identified (Supplementary Material Figure S1). The culture media of all four cultivations (about 450 mL each) were then loaded into the Ni-agarose column and the elution fractions were analyzed by SDS-PAGE. As can be seen in Figure 5, hirudin HV1 could be isolated in various fractions of all four expression experiments, but in comparably low amounts. Almost no contamination was present in the elution fractions. The total yields of all preparations are provided in Supplementary Material Table S4. 

For each clone and temperature, the fraction with the highest amount of hirudin HV1 was selected for further analyses. Dialysis, factor Xa protease treatment and removal of factor Xa protease were performed as described for the bacterial expression system. The complete removal of the His-tag was confirmed by subsequent SDS-PAGE analyses (see Supplementary Material Figure S2). The protein concentrations were determined, and the results are listed in Table 3.

### 3.5. Functional Characterization of Hirudin HV1 Expressed in P. pastoris GS115

**Figure 6:**
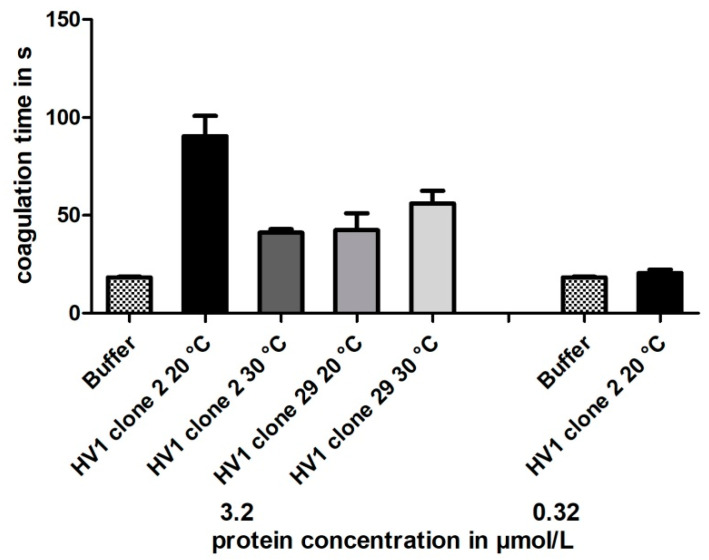
Thrombin time coagulation assays of recombinant hirudin HV1 preparations after cultivation and expression in *P. pastoris* GS115 clones 2 and 29 at either 20 or 30 °C, respectively. Results are means of three to six independent measurements.

As for hirudin HV1 and HLF1 expressed in *E. coli* strains SHuffle T7^®^ and DH5α (see above), the purified hirudin HV1 preparations that were expressed in *P. pastoris* GS115 clones 2 and 29 at either 20 or 30 °C of cultivation temperature were functionally tested for their thrombin-inhibitory potencies using the TT-test. The results are summarized in Figure 6.

All yeast-based hirudin HV1 preparations displayed thrombin-inhibitory potencies, but even at the high final concentration of 3.2 µmol/L, the clotting times were markedly less prolonged compared to the hirudin HV1 preparations upon bacterial expression (see Figure 3 and Figure 4). In addition, no clear effect of different cultivation temperatures could be observed.

## 4. Discussion

Recombinant proteins are expressed in heterologous hosts like bacteria, yeast, and insect or mammalian cells to obtain larger quantities of the “protein of interest” for scientific, therapeutic or commercial purposes [34,35]. However, many potential pitfalls exist and the final success critically depends on the choice of the right host expression system [36,37,38]. The correct disulfide bond formation is one of the most critical steps [39,40,41]. For the heterologous expression in bacteria, two strategies have been developed to enhance the correct formation of disulfide bonds in recombinant proteins: (i) strains that carry knock outs of the disulfide bond-reducing enzymes like thioredoxin reductase (ΔtrxB) and glutathione reductase (Δgor) genes (e.g., Origami of Novagen) or (ii) strains that co-express proteins that catalyze oxidative protein folding [42,43]. Disco (cytoplasmic disulfide bond formation in *E. coli*) is an example of the second strategy [44,45,46]. The *E. coli* strain SHuffle^®^ T7 carries the thioredoxin and glutathione reductases double knock-out, but additionally expresses a disulfide bond isomerase, DsbC [29]. A systematic comparison revealed that both strategies to enhance the correct formation of disulfide bonds in recombinant proteins have advantages and disadvantages [47].

The leech-derived thrombin inhibitor hirudin has not yet been expressed in one or the other of the improved *E. coli* strains. So far, all our attempts to express recombinant hirudins or hirudin-like factors (HLFs) were performed in *E. coli* strain DH5α that was cultivated at 37 °C, and the preparations of recombinant hirudins displayed high thrombin-inhibitory potencies (e.g., [13,48]). On the other hand, the *E. coli* strain SHuffle^®^ T7 was already used to successfully express the recombinant human cysteine-rich proteins cathepsin B and L [49]. Both hirudin HV1 and HLF1V of *H. medicinalis* were expressed in parallel in *E. coli* strains DH5α and SHuffle^®^ T7 at 30 °C, the optimal cultivation temperature of strain SHuffle^®^ T7. The functional characterization revealed that the preparations of both recombinant hirudin HV1 and HLF1V in *E. coli* SHuffle^®^ T7 displayed high thrombin-inhibitory potencies (see Figure 3). In contrast, the preparations in *E. coli* strain DH5α were considerably less inhibitory potent, not only compared to SHuffle^®^ T7 (see Figure 3) but also to previous preparations in DH5α [10]. This observation raised the question of whether the effect was due to the temperature or due to the genetic background of the strain. In a second experiment, we hence cultivated both *E. coli* strain DH5α and *E. coli* strain SHuffle^®^ T7 at temperatures of 22, 30, 37 and 42 °C, respectively, spanning large parts of the total growth range of *E. coli* [50]. The functional characterization of all preparations revealed clear optimum curves with either 37 °C (for *E. coli* strain DH5α) or 30 °C (for *E. coli* strain SHuffle^®^ T7) being the optimal cultivation temperatures to achieve maximal inhibitory potencies of recombinant hirudin HV1 (see Figure 4A,B). However, further variations (e.g., media composition, IPTG concentration or duration of protein expression [51]) may result in even higher inhibitory potencies of recombinant hirudin HV1 preparations.

It is very likely that the different inhibitory potencies of the respective hirudin HV1 preparations are due to differences in the correct formation of disulfide bonds. The presence of six cysteine residues within the amino acid sequence of hirudin HV1 results in a total of 76 different theoretically possible combinations of disulfide bond formation (calculated with the “Calculator of the number of possibilities for SS bridges in proteins”, [52]), and only one represents the correct form. Lower incubation temperatures generally favor the expression of soluble recombinant proteins in *E. coli* [53,54], but the influence of the cultivation temperature on the correct formation on disulfide bonds has not yet been thoroughly investigated.

Another promising strategy to enhance the output of a biologically active eukaryotic recombinant protein (e.g., hirudin HV1) is to switch from a bacterial to a eukaryotic expression system. The yeasts *P. pastoris* and *S. cerevisiae* represent such expression systems for hirudin [24,55,56,57]. Usually, recombinant hirudin expressed in yeast is secreted into the growth medium and eventually purified by gel filtration and anion exchange chromatography. We applied a slightly different strategy, however, that allowed for a direct comparison of the yeast expression system to the bacterial expression system. In general, the yield of hirudin HV1 was comparably low after yeast-based expression and did not substantially differ between the two selected clones (2 and 29) and the two incubation temperatures (20 and 30 °C, respectively; see Figure 5, Table 2 and Table S3). But even more importantly, the thrombin-inhibitory potencies of all yeast-expressed hirudin HV1 preparations were much lower compared to the bacterially expressed preparations (compare Figure 3, Figure 4 and Figure 6). N-glycan hyper-mannosylation is a post-translational modification that can occur in both *S. cerevisiae* and *P. pastoris* and may result in the expression of inactive recombinant proteins [58,59]. Since we have not deglycosylated our eukaryotic hirudin HV1 preparations, an inhibitory effect of hyper-glycosylation hence cannot be completely ruled out. However, the expression of hirudin variant HV2 in *P. pastoris* by Rosenfeld et al. [24] led to the purification of a recombinant hirudin that displayed a *Ki* value comparable to the *Ki* value of the bacterially expressed recombinant hirudin, indicating that hyper-glycosylation was not an issue. Interestingly, hirudin P6 of the Asian buffalo leech *Hirudinaria manillensis* Lesson (1842) is a naturally occurring Thr43-O-glycosylated and Tyr63-sulfated hirudin variant [60,61]. Glycosylated forms of hirudin P6 displayed lower *Ki* values compared with the unmodified protein but strikingly, the sulfated and deglycosylated form displayed the lowest Ki value [62].

Temperature is another factor that might influence the expression of recombinant proteins in yeast, and sub-physiological temperatures (20 instead of 30 °C) did improve the expression and assembly of difficult-to-express heterologous proteins in *S. cerevisiae* [63]. However, the cultivation of *P. pastoris* at 20 °C improved neither the yield nor the inhibitory potencies of the respective hirudin HV1 preparations in our study (see Figure 6 and Table 2).

## 5. Summary

We have expressed recombinant hirudin HV1 and HLF1V in two different bacterial (both factors) and one eukaryotic (hirudin HV1 only) expression systems and subsequently purified, processed and functionally characterized the expressed recombinant proteins in terms of their thrombin-inhibitory potencies. For the bacterial strains, the choice of an *E. coli* strain that was particularly designed for the enhanced formation of disulfide bonds in cysteine-containing proteins (namely *E. coli* strain SHuffle^®^ T7) resulted in the expression of a hirudin HV1 preparation with a moderately higher thrombin-inhibitory potency, compared with expression in a standard strain (namely *E. coli* strain DH5α), when both strains were cultivated at their optimal cultivation temperature of 30 °C (SHuffle^®^ T7) or 37 °C (DH5α), respectively. However, the expression of hirudin HV1 in both *E. coli* strains at different temperatures ranging from 22 to 42 °C led to preparations of the recombinant protein that in both cases exhibited a clear temperature-dependent optimum-curve of the inhibitory potencies: highest at 37 °C for *E. coli* DH5α and 30 °C for *E. coli* SHuffle^®^ T7, respectively, but lower at cultivation temperatures above the optimum and lowest at 22 °C. The correct formation of disulfide bonds is hence a function of the optimal cultivation temperature of the bacterial host, at least for *E. coli* strains DH5α and SHuffle^®^ T7 in combination with the expression of recombinant hirudin HV1. The expression of hirudin HV1 in the yeast *P. pastoris*, however, did result in preparations that exhibited only comparably low thrombin-inhibitory potencies. Hyper-glycosylation might be an explanation for that observation. Taken together, both *E. coli* strains DH5α and SHuffle^®^ T7 represents appropriate expression systems for hirudin variants (and very likely for other cysteine-rich leech-derived factors like decorsins and ornatins too) as long as the strains are cultivated at their optimal temperatures.

## Figures and Tables

**Table 1:** Total yields in mg/L of recombinant hirudin HV1 and HLF1V after expression in *E. coli* strains SHuffle^®^ T7 and DH5α at a cultivation temperature of 30 °C.

Expression System	Hirudin HV1	HLF1V
SHuffle^®^ T7	17.6	11.5
DH5α	21.2	13.8

**Table 2:** Concentrations of processed recombinant hirudin HV1 and HLF1V preparations after expression in *E. coli* strains SHuffle^®^ T7 and DH5α at a cultivation temperature of 30 °C.

Expression System	Hirudin HV1	HLF1V
SHuffle^®^ T7	132.4 µmol/L (931 µg/mL)	154.9 µmol/L (1011 µg/mL)
DH5α	190.9 µmol/L (1341 µg/mL)	128.2 µmol/L (842 µg/mL)

**Table 3:** Concentrations of processed recombinant hirudin HV1 after expression in *P. pastoris* GS115 clone 2 and clone 29 at 20 or 30 °C, respectively.

Cultivation Temperature	Clone 2	Clone 29
20 °C	59.3 µmol/L (416 µg/mL)	50.7 µmol/L (356 µg/mL)
30 °C	11.3 µmol/L (79 µg/mL)	34.3 µmol/L (241 µg/mL)

## Data Availability

All the data obtained in this study are available either in the article or in the Supplementary Material.

## Supplementary Materials

The following supporting information can be downloaded at: https://www.mdpi.com/article/10.3390/mps8040089/s1, Figure S1: SDS-PAGE analysis of cultivation medium, whole-cell lysates as well as supernatants and pellets after sonication of *P. pastoris* GS115 clones 2 and 29 after expression of hirudin HV1 and cultivation at 30 °C; Figure S2: Factor Xa protease treatment of selected elution fractions (ef) of hirudin HV1 expressed in *P. pastoris* GS115 clones 2 and 29 cultivated at 30 °C (A) or 20 °C (B); Table S1: Optical densities (OD_600_) of *E. coli* strains DH5α and SHuffle^®^ T7 at start/at harvesting during expression of recombinant hirudin HV1 at different cultivation temperatures; Table S2: Total yields in mg/L of recombinant hirudin HV1 after expression in *E. coli* strains DH5α and SHuffle^®^ T7 at different cultivation temperatures; Table S3: Concentrations of processed recombinant hirudin HV1 after expression in *E. coli* strains DH5α and SHuffle^®^ T7 at different cultivation temperatures; Table S4: Total yields in mg/L of recombinant hirudin HV1 after expression in *P. pastorisi* clones 2 and 29 at different cultivation temperatures.

## References

[1] Dodt J., Müller H.-P., Seemüller U., Chang J.-Y. (1984). The complete amino acid sequence of hirudin, a thrombin specific inhibitor: Application of colour carboxymethylation. FEBS Lett..

[2] Markwardt F. (1970). Hirudin as an inhibitor of thrombin. Method. Enzym..

[3] Markwardt F. (1989). Development of hirudin as an antithrombotic agent. Semin. Thromb. Hemost..

[4] Johnson P.H. (1994). Hirudin: Clinical potential of a thrombin inhibitor. Annu. Rev. Med..

[5] Greinacher A., Warkentin T.E. (2008). The direct thrombin inhibitor hirudin. Thromb. Haemost..

[6] Dodt J., Seemüller U., Maschler R., Fritz H. (1985). The complete covalent structure of hirudin. Localization of the disulfide bonds. Biol. Chem. Hoppe Seyler.

[7] Rydel T.J., Ravichandran K.G., Tulinsky A., Bode W., Huber R., Roitsch C., Fenton J.W. (1990). The structure of a complex of recombinant hirudin and human alpha-thrombin. Science.

[8] Rydel T.J., Tulinsky A., Bode W., Huber R. (1991). Refined structure of the hirudin-thrombin complex. J. Mol. Biol..

[9] Warkentin T.E. (2004). Bivalent direct thrombin inhibitors: Hirudin and bivalirudin. Best. Pract. Res. Clin. Haematol..

[10] Müller C., Lukas P., Böhmert M., Hildebrandt J.-P. (2020). Hirudin or hirudin-like factor—That is the question: Insights from the analyses of natural and synthetic HLF variants. FEBS Lett..

[11] Harvey R.P., Degryse E., Stefani L., Schamber F., Cazenave J.P., Courtney M., Tolstoshev P., Lecocq J.P. (1986). Cloning and expression of a cDNA coding for the anticoagulant hirudin from the bloodsucking leech, *Hirudo medicinalis*. Proc. Nat. Acad. Sci. USA.

[12] Dodt J., Machleidt W., Seemüller U., Maschler R., Fritz H. (1986). Isolation and characterization of hirudin isoinhibitors and sequence analysis of hirudin PA. Biol. Chem. Hoppe Seyler.

[13] Müller C., Mescke K., Liebig S., Mahfoud H., Lemke S., Hildebrandt J.-P. (2016). More than just one: Multiplicity of Hirudins and Hirudin-like Factors in the Medicinal Leech, *Hirudo medicinalis*. Mol. Genet. Genom..

[14] Müller C., Haase M., Lemke S., Hildebrandt J.-P. (2017). Hirudins and hirudin-like factors in *Hirudinidae*: Implications for function and phylogenetic relationships. Parasitol. Res..

[15] Müller C., Lukas P., Sponholz D., Hildebrandt J.-P. (2020). The hirudin-like factors HLF3 and HLF4- hidden hirudins of European medicinal leeches. Parasitol. Res..

[16] Ben Ahmed R., Abilov A., Müller C. (2024). Diversity of hirudin and hirudin-like factor genes in the North-African medicinal leech, *Hirudo troctina*. Parasitol. Res..

[17] Abdualkader A.M., Ghawi A.M., Alaama M., Awang M., Merzouk A. (2013). Leech therapeutic applications. Indian J. Pharm. Sci..

[18] Sig A.K., Guney M., Guclu A.U., Ozmen E. (2017). Medicinal leech therapy—An overall perspective. Integr. Med. Res..

[19] Dong H., Ren J.-X., Wang J.-J., Ding L.-S., Zhao J.-J., Liu S.-Y., Gao H.-M. (2016). Chinese medicinal leech: Ethnopharmacology, phytochemistry, and pharmacological activities. Evid. Based Complement. Alternat. Med..

[20] Qiu J., Lingna W., Jinghong H., Yongqing Z. (2019). Oral administration of leeches (Shuizhi): A review of the mechanisms of action on antiplatelet aggregation. J. Ethnopharmacol..

[21] Greinacher A., Völpel H., Janssens U., Hach-Wunderle V., Kemkes-Matthes B., Eichler P., Mueller-Velten H.G., Pötzsch B. (1999). Recombinant hirudin (lepirudin) provides safe and effective anticoagulation in patients with heparin-induced thrombocytopenia: A prospective study. Circulation.

[22] Lubenow N., Greinacher A. (2002). Hirudin in heparin-induced thrombocytopenia. Semin. Thromb. Hemost..

[23] Riehl-Bellon N., Carvallo D., Acker M., Van Dorsselaer A., Marquet M., Loison G., Lemoine Y., Brown S.W., Courtney M., Roitsch C. (1989). Purification and biochemical characterization of recombinant hirudin produced by *Saccharomyces cerevisiae*. Biochemistry.

[24] Rosenfeld S.A., Nadeau D., Tirado J., Hollis G.F., Knabb R.M., Jia S. (1996). Production and purification of recombinant hirudin expressed in the methylotrophic yeast *Pichia pastoris*. Protein Expr. Purif..

[25] Kochanowski R., Kotłowski R., Szweda P. (2006). Novel method of expression and purification of hirudin based on pBAD TOPO, pTYB12 vectors and gene synthesis. Protein Expr. Purif..

[26] Nowak G. (2002). Pharmacology of recombinant hirudin. Semin. Thromb. Hemost..

[27] Wüstenhagen D.A., Lukas P., Müller C., Aubele S.A., Hildebrandt J.-P., Kubick S. (2020). Cell-free synthesis of the hirudin variant 1 of the blood-sucking leech *Hirudo medicinalis*. Sci. Rep..

[28] Hanahan D., Glover D.M. (1985). Techniques for transformation of *E. coli*. DNA Cloning: A Practical Approach.

[29] Lobstein J., Emrich C.A., Jeans C., Faulkner M., Riggs R., Berkmen M. (2012). SHuffle, a novel *Escherichia coli* protein expression strain capable of correctly folding disulfide bonded proteins in its cytoplasm. Microb. Cell Fact..

[30] Green M., Sambrook J. (2012). Molecular Cloning: A Laboratory Manual.

[31] Gill S.C., von Hippel P.H. (1989). Calculation of protein extinction coefficients from amino acid sequence data. Anal. Biochem..

[32] Pace C.N., Vajdos F., Fee L., Grimsley G., Gray T. (1995). How to measure and predict the molar absorption coefficient of a protein. Protein Sci..

[33] Zhong Y., Yang L., Guo Y., Fang F., Wang D., Li R., Jiang M., Kang W., Ma J., Sun J. (2014). High-temperature cultivation of recombinant *Pichia pastoris* increases endoplasmic reticulum stress and decreases production of human interleukin-10. Microb. Cell Fact..

[34] di Leandro L., Colasante M., Pitari G., Ippoliti R. (2023). Hosts and heterologous expression strategies of recombinant toxins for therapeutic purposes. Toxins (Basel).

[35] Jayakrishnan A., Rosli W.R.W., Tahir A.R.M., Razak F.S.A., Kee P.E., Ng H.S., Chew Y.-L., Lee S.-K., Ramasamy M., Tan C.S. (2024). Evolving paradigms of recombinant protein production in pharmaceutical industry: A rigorous review. Sci..

[36] Fernández F.J., Vega M.C., Vega M. (2016). Choose a suitable expression host: A survey of available protein production platforms. Advanced Technologies for Protein Complex Production and Characterization. Advances in Experimental Medicine and Biology.

[37] Gomes A.R., Byregowda S.M., Veeregowda B.M., Balamurugan V. (2016). An overview of heterologous expression host systems for the production of recombinant proteins. Adv. Anim. Vet. Sci..

[38] Schütz A., Bernhard F., Berrow N., Buyel J.F., Ferreira-da-Silva F., Haustraete J., van den Heuvel J., Hoffmann J.-E., de Marco A., Peleg Y. (2023). A concise guide to choosing suitable gene expression systems for recombinant protein production. STAR Protoc..

[39] de Marco A. (2009). Strategies for successful recombinant expression of disulfide bond-dependent proteins in *Escherichia coli*. Microb. Cell Fact..

[40] Berkmen M. (2012). Production of disulfide-bonded proteins in *Escherichia coli*. Protein Expr. Purif..

[41] Pouresmaeil M., Azizi-Dargahlou S. (2023). Factors involved in heterologous expression of proteins in *E. coli* host. Arch. Microbiol..

[42] Saaranen M.J., Ruddock L.W. (2019). Applications of catalyzed cytoplasmic disulfide bond formation. Biochem. Soc. Trans..

[43] Ferrer-Miralles N., Garcia-Fruitós E. (2024). Heterologous expression of difficult to produce proteins in bacterial systems. Int. J. Mol. Sci..

[44] Hatahet F., Nguyen V.D., Salo K.E.H., Ruddock L.W. (2010). Disruption of reducing pathways is not essential for efficient disulfide bond formation in the cytoplasm of *E. coli*. Microb. Cell. Fact..

[45] Nguyen V.D., Hatahet F., Salo K.E.H., Enlund E., Zhang C., Ruddock L.W. (2011). Pre-expression of a sulfhydryl oxidase significantly increases the yields of eukaryotic disulfide bond containing proteins expressed in the cytoplasm of *E.coli*. Microb. Cell Fact..

[46] Gąciarz A., Veijola J., Uchida Y., Saaranen M.J., Wang C., Hörkkö S., Ruddock L.W. (2016). Systematic screening of soluble expression of antibody fragments in the cytoplasm of *E. coli*. Microb. Cell Fact..

[47] Castillo-Corujo A., Uchida Y., Saaranen M.J., Ruddock L.W. (2024). *Escherichia coli* cytoplasmic expression of disulfide-bonded proteins: Side-by-side comparison between two competing strategies. J. Microbiol. Biotechnol..

[48] Müller C., Wang Z., Hamann M., Sponholz D., Hildebrandt J.-P. (2022). Life without blood: Molecular and functional analysis of hirudins and hirudin-like factors of the Asian non- hematophagous leech *Whitmania pigra*. J. Thromb. Haemost..

[49] Möller C., Rimkus N., Skala F.F.O., Merouze M., Böttcher D., Dörr M., Bornscheuer U.T. (2024). Improved recombinant expression of soluble cathepsin B and L in *Escherichia coli*. Appl. Microbiol. Biotechnol..

[50] Tuttle A.R., Trahan N.D., Son M.S. (2021). Growth and maintenance of *Escherichia coli* laboratory strains. Curr. Protoc..

[51] Francis D.M., Page R. (2010). Strategies to optimize protein expression in *E. coli*. Curr. Protoc. Protein Sci..

[52] Combet C., Blanchet C., Geourjon C., Deléage G. (2000). NPS@: Network protein sequence analysis. Trends Biochem. Sci..

[53] Schein C., Noteborn M. (1988). Formation of soluble recombinant proteins in *Escherichia coli* is favored by lower growth temperature. Nat. Biotechnol..

[54] San-Miguel T., Pérez-Bermúdez P., Gavidia I. (2013). Production of soluble eukaryotic recombinant proteins in *E. coli* is favoured in early log-phase cultures induced at low temperature. Springerplus.

[55] Huang Y., Zhang Y., Wu Y., Wang J., Liu X., Dai L., Wang L., Yu M., Mo W. (2012). Expression, purification, and mass spectrometric analysis of 15N, 13C-labeled RGD-hirudin, expressed in *Pichia pastoris*, for NMR studies. PLoS ONE.

[56] Sohn J.-H., Lee S.-K., Choi E.-S., Rhee S.-K. (1991). Gene expression and secretion of the anticoagulant hirudin in *Saccharomyces cerevisiae*. J. Microbiol. Biotechnol..

[57] Kim M.D., Rhee S.K., Seo J.H. (2001). Enhanced production of anticoagulant hirudin in recombinant *Saccharomyces cerevisiae* by chromosomal delta-integration. J. Biotechnol..

[58] Jayaraj R., Smooker P.M. (2009). So you need a protein—A guide to the production of recombinant proteins. Open Vet. Sci. J..

[59] Kastberg L.L.B., Ard R., Jensen M.K., Workman C.T. (2022). Burden imposed by heterologous protein production in two major industrial yeast cell factories: Identifying sources and mitigation strategies. Front. Fungal. Biol..

[60] Steiner V., Knecht R., Gruetter M., Raschdorf F., Gassmann E., Maschler R. (1990). Isolation and purification of novel hirudins from the leech *Hirudinaria manillensis* by high-performance liquid chromatography. J. Chromatogr..

[61] Steiner V., Knecht R., Börnsen K.O., Gassmann E., Stone S.R., Raschdorf F., Schlaeppi J.M., Maschler R. (1992). Primary structure and function of novel O-glycosylated hirudins from the leech *Hirudinaria manillensis*. Biochemistry.

[62] Hsieh Y.S.Y., Wijeyewickrema L.C., Wilkinson B.L., Pike R.N., Payne R.J. (2014). Total synthesis of homogeneous variants of hirudin P6: A post-translationally modified anti-thrombotic leech- derived protein. Angew. Chem. Int. Ed. Engl..

[63] So K.-K., Le N.M.T., Nguyen N.-L., Kim D.-H. (2023). Improving expression and assembly of difficult- to-express heterologous proteins in *Saccharomyces cerevisiae* by culturing at a sub-physiological temperature. Microb. Cell Fact..

