# Supplementary material for: Expression of Recombinant Hirudin in Bacteria and Yeast: A Comparative Approach
Source: Methods Protoc. 2025 Aug 3;8(4):89. doi: 10.3390/mps8040089 (PMC12388516; doi:10.3390/mps8040089)
Supplement: Supplementary file 1 [file mps-08-00089-s001.zip › mps-3713621-supplementary.pdf]

Supplementary Materials

Figure S1

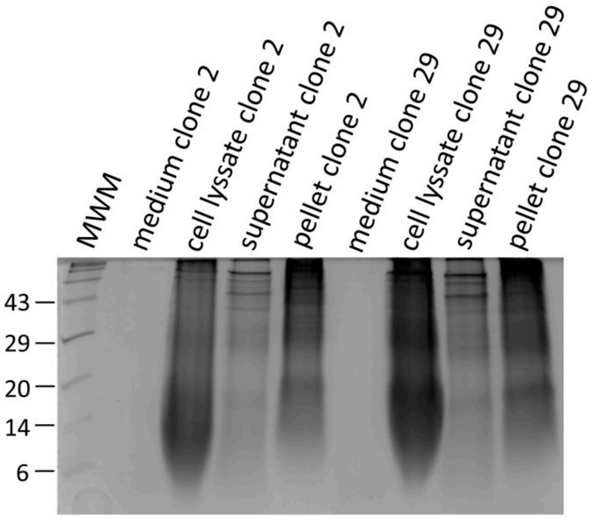

Figure S1: SDS-PAGE analysis of cultivation medium, whole-cell lysates, supernatants and pellets after sonication of *P. pastoris* GS115 clones 2 and 29 after expression of hirudin HV1 and cultivation at 30 °C. The numbers on the left side indicate the molecular masses in kDa of the respective molecular weight marker (MWM) bands.

Figure S2

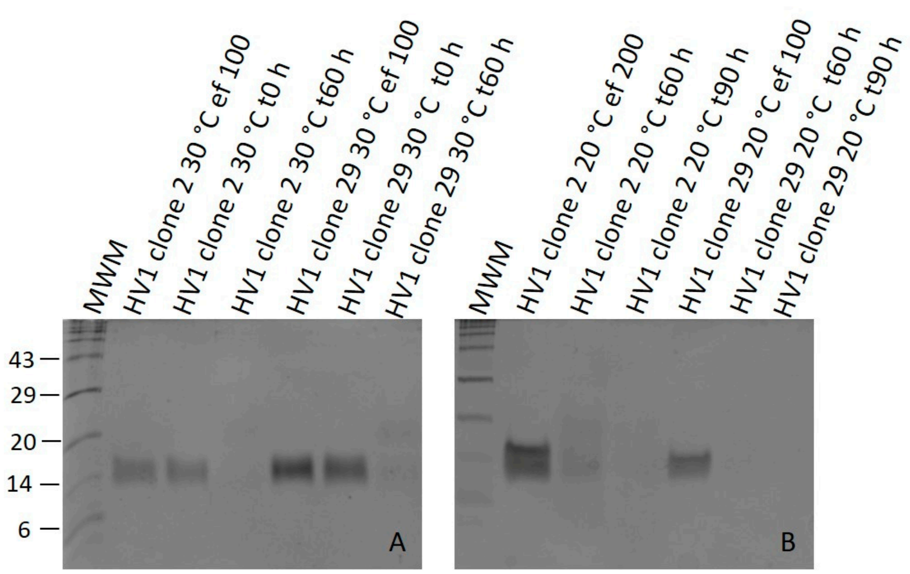

Figure S2: Factor Xa protease treatment of selected elution fractions (ef) of hirudin HV1 expressed in *P. pastoris* GS115 clones 2 and 29 cultivated at 30 °C (A) or 20 °C (B). The numbers on the left side indicate the molecular mass in kDa of the respective molecular weight marker (MWM) bands.
Please note that untagged hirudin HV1 and HLF1V are very poorly stained by Coomassie brilliant blue stain due to the low number (hirudin HV1) of basic amino acid residues in the molecules, whereas the His-tagged proteins are properly stained due to the His-tag having not only six histidine residues, but also two additional basic amino acid residues.

Table S1: Optical densities (OD_{600}) of *E. coli* strains DH5α and SHuffle® T7 at start/at harvesting during expression of recombinant hirudin HV1 at different cultivation temperatures.

Cultivation temperature	DH5α	SHuffle® T7
22 °C	0,48 / 0,76	0,55 / 1,18
30 °C	0,49 / 0,95	0,55 / 1,42
37 °C	0,55 / 1,10	0,51 / 1,23
42 °C	0,48 / 1,04	0,54 / 1,18

Table S2: Total yields in mg/l of recombinant hirudin HV1 after expression in *E. coli* strains DH5α and SHuffle® T7 at different cultivation temperatures.

Cultivation temperature	DH5α	SHuffle® T7
22 °C	14,0	24,0
30 °C	24,0	34,8
37 °C	28,4	34,1
42 °C	18,3	41,5

Table S3: Concentrations of processed recombinant hirudin HV1 after expression in *E. coli* strains DH5α and SHuffle® T7 at different cultivation temperatures.

Cultivation temperature	DH5α	SHuffle® T7
22 °C	109.7 µmol/L (771 µg/mL)	175.3 µmol/L (1232 µg/mL)
30 °C	148.8 µmol/L (1046 µg/mL)	166.0 µmol/L (1166 µg/mL)
37 °C	184.5 µmol/L (1296 µg/mL)	210.5 µmol/L (1479 µg/mL)
42 °C	156.3 µmol/L (1098 µg/mL)	205.4 µmol/L (1443 µg/mL)

Table S4: Total yields in mg/L of recombinant hirudin HV1 after expression in *P. pastorisi* clones 2 and 29 at different cultivation temperatures.

Cultivation temperature	Clone 2	Clone 29
20 °C	6,5	8,1
30 °C	10,0	6,4
